# Histoacryl-Kleber zur Akutversorgung von Hornhautperforationen bei nekrotisierender herpetischer Keratitis

**DOI:** 10.1007/s00347-020-01284-2

**Published:** 2020-12-09

**Authors:** M. El Halabi, B. Seitz, A. Quintin, S. Suffo, F. Flockerzi, U. Schlötzer-Schrehardt, L. Daas

**Affiliations:** 1 Klinik für Augenheilkunde, Universitätsklinikum des Saarlandes UKS, Kirrberger Str. 100, Gebäude 22, 66421 Homburg/Saar, Deutschland; 2 Institut für Pathologie, Universitätsklinikum des Saarlandes UKS, Homburg/Saar, Deutschland; 3 Augenklinik, Universität Erlangen-Nürnberg, Erlangen, Deutschland

Das Herpes-simplex-Virus (HSV) ist eine der häufigsten Ursachen einer infektiösen Keratitis. Die herpetische Keratitis kann als 1. epitheliale Keratitis (dendritica), 2. stromale Keratitis (nekrotisierend vs. nicht nekrotisierend = „interstitiell“), 3. Endotheliitis (=„disziforme Keratitis“), 4. neurotrophe Keratopathie (=sog. „metaherpetische Keratitis“) oder 5. (vaskularisierte) Hornhautnarben vorliegen [1, 2]; 40 % der herpetischen Keratitis sind stromal (nekrotisierend 7 %, nicht nekrotisierend 93 %) [3]. Bei ulzerierender nekrotisierender stromaler Keratitis finden sich im Stroma im Bereich des Ulkus aktive und sich replizierende Viren [1, 4]. Typischerweise tritt der Befund einseitig auf, und die Hornhautsensibilität ist deutlich herabgesetzt. Es lassen sich virale Anteile des Genoms sowie virale Antigene im befallenen Hornhautgewebe nachweisen. Bei verzögerter Abheilung und Rezidiven entstehen oft dichte Narben, Gewebeverlust und Neovaskularisation. Unbehandelt ist das Perforationsrisiko eines Ulkus herpetischer Genese sehr groß [1]. Die Hornhautperforation kann zu gravierendem Sehverlust, aber auch zum Verlust des Auges führen [5].

Die Ursachen der Hornhautperforation werden in 2 Kategorien unterteilt: 25 % infektiös (herpetisch, bakteriell, mykotisch) und 75 % nicht infektiös [5–7]. Zu den Ursachen der nicht infektiösen Keratopathie gehören das Einschmelzen der Hornhaut bei rheumatischer Arthritis, ein schweres trockenes Auge, Lagophthalmus und das neurotrophe Ulkus [6, 7]. Augen mit Hornhautperforation müssen sofort behandelt werden, um die anatomische Integrität der Hornhaut zu erhalten und Komplikationen wie eine Endophthalmitis und expulsive Aderhautblutung zu verhindern [8].

In dieser Triple-Kasuistik beschreiben wir Patienten, die nach Histoacryl-Kleber-Versorgung im Akutstadium final erfolgreich mittels perforierender Keratoplastik (PKP) versorgt wurden, und präsentieren histologische und elektronenmikroskopische Befunde.

## Falldarstellungen

### Patient 1

#### Anamnese.

Ein 62-jähriger Patient wurde erstmals 2016 in unsere Klinik überwiesen, mit der Fragestellung nach PKP bei Zustand nach perforiertem Hornhautulkus herpetischer Genese im Jahr 2007, welches dann mit Histoacryl-Kleber extern versorgt wurde. Bei dem Patienten bestanden anamnestisch keine Allgemeinerkrankungen.

#### Klinischer Befund.

**Figure 1:**
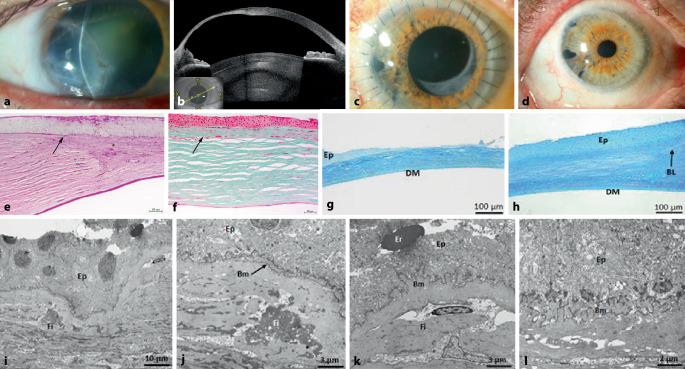

Der bestkorrigierte Dezimalvisus betrug 0,16 am betroffenen RA. Der Augeninnendruck (IOD) lag bei 14 mm Hg. Spaltlampenbiomikroskopisch zeigten sich eine großflächige temporale (mittel)periphere bis zur optischen Achse reichende tiefe stromale Vernarbung mit oberflächlicher und tiefer Vaskularisation sowie eine extreme Stromaverdünnung bis zum Limbus. Die Vorderkammer war reizfrei (Abb. 1a). Des Weiteren zeigte sich eine Cataracta corticonuclearis. Der Fundus war unauffällig.

#### Diagnostik.

Bei der optischen Kohärenztomographie des vorderen Augenabschnittes ergab sich am betroffenen Auge temporal inferior eine extreme Stromaverdünnung (Hornhaut in der Pachymetrie an ihrer dünnsten Stelle noch 211 µm dick) (Abb. 1b). In der Pentacam zeigte sich ein hoher irregulärer Astigmatismus der kornealen Vorderfläche von 11,1 dpt.

#### Therapie und Verlauf.

Die initiale Therapie wurde mit Dexpanthenol-Augengel (AG) 5‑mal/Tag, Dexpanthenol-Augensalbe (AS) 1‑mal/Tag zur Nacht, Dexamethason 1,0 mg/ml-Augentropfen (AT) 1‑mal/Tag morgens und Ciclosporin 1 mg/ml (Ikervis)-AT zur Nacht am betroffenen Auge eingeleitet. Nach Verfügbarkeit eines Hornhautgewebes führten wir rechts eine elektive exzentrische (nach temporal dezentrierte) *PKP (Handtrepan)* mit einem großen Transplantatdurchmesser (Durchmesser Öffnung/Transplantat = 10,0/10,5 mm) als „triple procedure“ durch (Abb. 1c). Das Transplantat wurde mit 32 Einzelknüpfnähten befestigt. Sechs Wochen nach der Entfernung aller Hornhautfäden betrug der bestkorrigierte Dezimalvisus 0,6, und es zeigte sich klinisch eine klare Hornhaut ohne Hinweis auf eine Abstoßungsreaktion (Abb. 1d). Intraoperativ wurde das Hornhautgewebe zur histologischen und transmissionselektronenmikroskopischen Untersuchung geschickt, und es zeigte sich eine regenerierte Epithelzellschicht mit Verlust der Bowman-Lamelle und vaskularisierter, subepithelial betonter Hornhautstromanarbe (Abb. 1e, f), mit Nachweis von Fibrinaggregaten im Stromabereich (Abb. 1g–l). Eine Dauertherapie mit Prednisolon AT‑0,12 % 2‑mal/Tag, und Virgan-AG 1‑mal/Tag wurde rechts empfohlen. Die systemische postoperative Therapie bestand aus Mycophenolat-Mofetil-Tabletten (Tbl.) 720 mg 2‑mal/Tag als Immunsuppression (aufgrund des großen TDMs) und einer antiviralen systemischen Therapie mittels Aciclovir-Tbl. 400 mg 5‑mal/Tag für 6 Wochen postoperativ, dann 2‑mal/Tag für 1 Jahr.

### Patient 2

#### Anamnese.

Ein 46-jähriger Patient mit bekannter atopischer Dermatitis stellte sich 2018 notfallmäßig bei uns mit der Fragestellung nach PKP à chaud bei Zustand nach Histoacryl-Kleber und Kontaktlinse (extern) bei perforiertem Hornhautulkus herpetischer Genese vor 14 Tagen vor. Keratokonus war bei dem Patienten seit 1998 bekannt, aber ohne Progressionszeichen.

#### Klinischer Befund.

**Figure 2:**
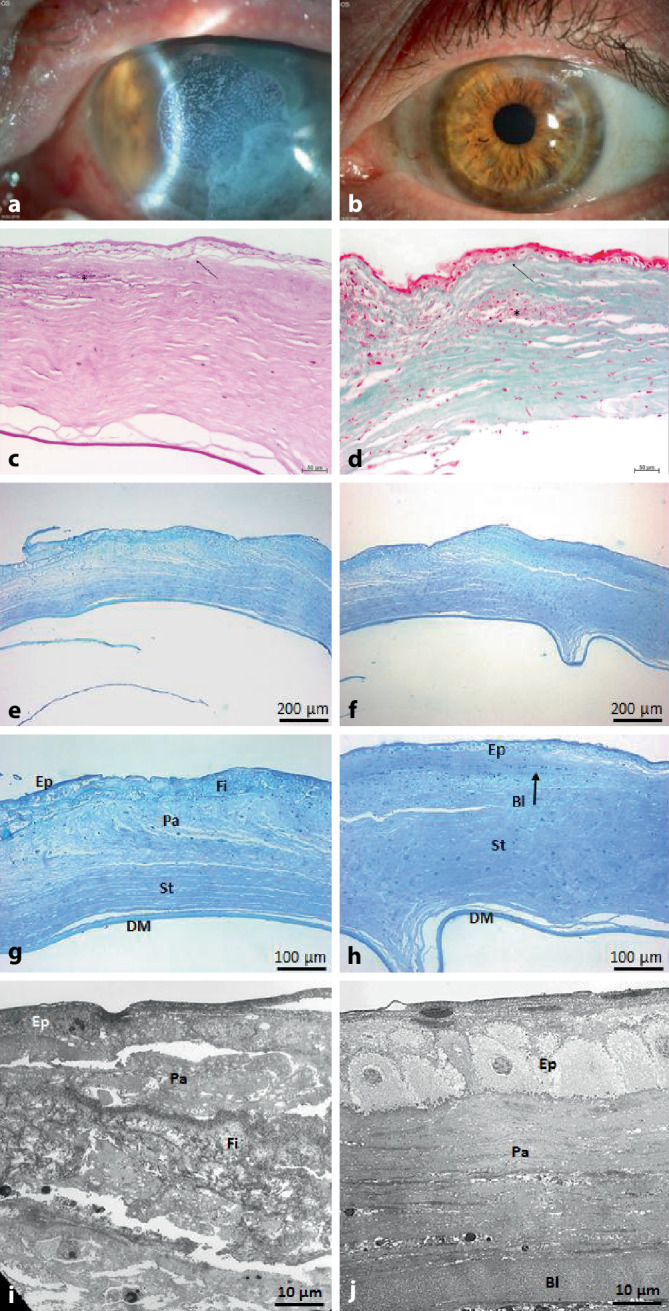

Der Visus betrug Handbewegung am betroffenen linken Auge (LA). Der IOD war palpatorisch normoton. Spaltlampenbiomikroskopisch zeigte sich links ein perforiertes, zentrales Hornhautulkus, auf das extern vor 14 Tagen eine harte Kontaktlinse und Histoacryl geklebt worden war. Darüber lag eine weiche Verbandskontaktlinse (Abb. 2a). Des Weiteren zeigte sich eine Cataracta matura. Ultrasonographisch lag die Netzhaut an.

#### Therapie und Verlauf.

Wir führten eine zentrierte *Excimerlaser-PKP à chaud* mit TDM von 8,0/8,1 mm als „triple procedure“ durch. Das Transplantat wurde mit 24 Einzelknüpfnähten befestigt. Zwei Jahre postoperativ (nach der Entfernung aller Hornhautfäden) betrug der bestkorrigierte Dezimalvisus 0,6 und zeigte klinisch eine klare Hornhaut ohne Hinweis auf eine Abstoßungsreaktion (Abb. 2b). In der Histologie sowie in der Transmissionselektronenmikroskopie zeigte sich eine verschmälerte Epithelzellschicht mit Verlust der Bowman-Lamelle und Ablagerung von Fibrinaggregaten im Ulkusbereich (Abb. 2c–j). Eine Dauertherapie mit Prednisolon-AT 0,12 % 2‑mal/Tag, und Virgan-AG 1‑mal/Tag rechts sowie eine postoperative Therapie mit Ciclosporin-Tbl. 150 mg 2‑mal/Tag als Immunsuppression (bei atopischer Dermatitis) und eine antivirale systemische Therapie mittels Aciclovir-Tbl. 400 mg 5‑mal/Tag für 6 Wochen, dann 2‑mal/Tag für 1 Jahr wurden empfohlen.

### Patient 3

#### Anamnese.

Ein 75-jähriger Patient stellte sich im Februar 2020 notfallmäßig bei uns mit der Fragestellung nach PKP à chaud bei Zustand nach Histoacryl-Kleber und Kontaktlinse (extern) bei perforiertem Hornhautulkus herpetischer Genese vor 7 Tagen vor.

#### Klinischer Befund.

**Figure 3:**
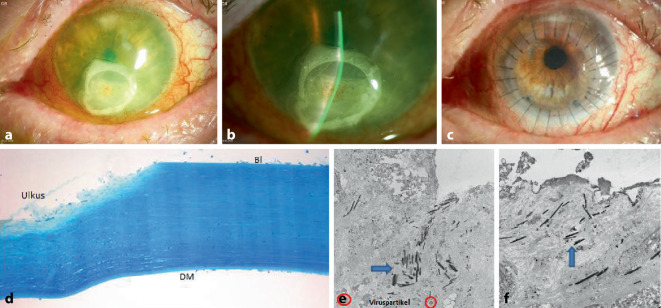

Der Visus betrug Handbewegung am betroffenen LA. Der IOD lag bei 6 mm Hg. Spaltlampenbiomikroskopisch zeigte sich links ein perforiertes inferiores Hornhautulkus, auf das extern vor 7 Tagen eine harte Kontaktlinse mit Histoacryl geklebt worden war. Darüber lag eine weiche Verbandskontaktlinse (Abb. 3a, b). Ultrasonographisch lag die Netzhaut an.

#### Therapie und Verlauf.

Wir führten eine exzentrische (nach inferior dezentrierte) *Excimerlaser-PKP à chaud* mit TDM von 8,5/8,6 mm durch. Das Transplantat wurde mit 26 Einzelknüpfnähten befestigt. Bei der Kontrolluntersuchung 4 Monate postoperativ betrug der bestkorrigierte Dezimalvisus 0,3, und es zeigte sich ein klares Transplantat mit festen Fäden und ohne Abstoßungszeichen (Abb. 3c, d). In der Transmissionselektronenmikroskopie zeigten sich nach einer durchgeführten Keratoplastik à chaud folgende Veränderungen: im Ulkusbereich degeneriertes Stroma, degenerierte Keratozyten mit Viruspartikeln, Granulozyten, oberflächliche nadelförmige kristalline Einlagerungen (evtl. Kleberreste), das Endothel fehlte (Abb. 3e, f). Postoperativ wurde eine Therapie mit Prednisolonacetat-AT 5‑mal/Tag, alle 8 Wochen um 1‑mal zu reduzieren, Moxifloxacin-AT 5‑mal/Tag für 5 Wochen, Ganciclovir-AG 5‑mal/Tag und Natriumhyaluronat-AG 5‑mal/Tag sowie systemisch mit Methylprednisolon-Tbl. 80 mg 1‑mal/Tag, alle 2 Tage um 20 mg zu reduzieren, und Aciclovir-Tbl. 400 mg 3‑mal/Tag wegen partieller Niereninsuffizienz eingeleitet.

## Diskussion

Die Hornhautperforation im Rahmen einer ulzerierenden nekrotisierenden stromalen Keratitis herpetischer Genese (sog. „herpetisches Ulkus“) ist eine ernsthafte Komplikation, die zum Verlust des Auges führen kann. Eine Hornhautperforation muss zeitnah verschlossen werden, um die kollabierte Vorderkammer wiederherzustellen und das Auge zu stabilisieren. Bei inadäquater Versorgung können ein irreversibles Winkelblockglaukom, eine mikrobielle Endophthalmitis oder gar eine expulsive Aderhautblutung auftreten [9]. Es gibt eine Vielzahl von Ansätzen für die Behandlung von Hornhautperforationen, von nichtchirurgischen Behandlungen wie therapeutische Kontaktlinsen mit/ohne Histoacryl-Kleber bis hin zu den chirurgischen Modalitäten wie einfache Hornhautnähte, mehrschichtige Amnionmembrantransplantation (AMT) und die tektonischen perforierenden Hornhauttransplantationen (PKP à chaud) (Tab. 1). Die Bindehautdeckung stellt heute die Ultima Ratio dar. Die Wahl der Behandlung hängt von der Größe und der Lage der Perforation sowie den zugrunde liegenden Erkrankungen ab [5, 6, 9].

**Table 1:** 

Therapieoptionen	Vorteile	Nachteile
*Therapeutische KL*	Leicht verfügbar	Oftmals unzureichende Abdichtung
*Hornhautnaht*	Leicht verfügbar	Nicht möglich bei großen Defekten durch Einschmelzung
*Histoacryl-Kleber*	Unterbricht Einschmelzungsprozess	Keine definitive Lösung
	Erlaubt elektive PKP im reizfreiem Intervall	
*AMT*	Tektonisch einfach	Die Vorderkammer formiert sich oft nicht > anteriore Synechien
*Kurative und/oder tektonische Keratoplastik*	Sofortige Stabilisierung des Bulbus	Hornhautspendergewebe nicht überall verfügbar
	Schnelle optische Rehabilitation	Technisch anspruchsvoll
*Bindehautdeckung*	In jeder Augenklinik möglich	Ultima Ratio, da oft keine anschließende Visusrehabilitation mehr möglich

*KL* Kontaktlinse, *AMT* Amnionmembrantransplantation

Im Jahr 1968 publizierten Refojo und Webster eine Pionierarbeit über die Verwendung von Gewebeklebern zur Behandlung von Hornhautperforationen [10]. Seitdem haben Forschungsstudien gezeigt, dass die direkte, frühe Applikation eines Klebstoffs auf das Ulkusbett und die angrenzende Basalmembran sowie eine Verbandskontaktlinse das fortschreitende Einschmelzen des Hornhautstromas unterbrechen können [11]. Dadurch wird die Reepithelisierung in der Zone des geschädigten und nackten Hornhautstromas verzögert. Infolgedessen wird die Migration der polymorphkernigen Leukozyten, die nachweislich eine starke proteolytische Aktivität haben, gehemmt [11, 12]. Die Unterbrechung des Einschmelzungsprozesses ist am erfolgreichsten, wenn sie früh im Verlauf angewendet wird, bevor sich eine überwältigende Anzahl von polymorphkernigen Leukozyten akkumuliert [5, 11].

Das Hornhautkleben stellt in Augenkliniken ohne Transplantationskompetenz eine valide Behandlungsoption dar [5, 11, 13]. Sie bietet eine tektonische Unterstützung und erhält vorübergehend die strukturelle Integrität des Bulbus. Darüber hinaus wird die Vorderkammer formiert, und zirkuläre vordere Synechien mit konsekutivem Winkelblockglaukom werden vermieden. Trotzdem wird eine elektive PKP zur visuellen Rehabilitation zu einem späteren Zeitpunkt erforderlich sein [11–13]. Die Verzögerung der perforierenden Keratoplastik durch den Einsatz von Hornhautkleber führt in der Regel nicht zu schlechteren Ergebnissen [13].

Ein idealer Gewebekleber sollte eine überlegene Zugfestigkeit haben, ungiftig und indirekt entzündungshemmend sein. Die Klebstoffe sind in 2 Hauptkategorien erhältlich: synthetische Klebstoffe (einschließlich Cyanacrylat- und Polyethylenglykol[PEG]-Derivate) und biologische Klebstoffe (wie Fibrin). Beide eignen sich am besten für kleine (<3 mm) Defekte [12, 14, 15].

Der biologische Fibrinkleber führt im Vergleich zu dem synthetischen Cyanacrylat-Kleber zu einer schnelleren Heilung und weniger Hornhautvaskularisierung, hat aber weniger Zugfestigkeit im Vergleich zu Cyanacrylat-Kleber [14]. Der Hauptnachteil biologischer Klebstoffe besteht darin, dass sie viel schneller abgebaut werden als synthetische Klebstoffe. Zusätzlich haben sie keine bakteriostatische Wirkung (wie synthetische Klebstoffe) [5, 13, 14]. Bei einer Hornhautperforation ist die Angabe über die Erfolgsrate in der Literatur sehr umstritten und variiert zwischen 22 und 73 % [13, 15].

In den Augenkliniken mit Transplantationskompetenz ist die PKP à chaud die bevorzugte Methode zur Behandlung des perforierten Hornhautulkus herpetischer Genese (Tab. 1). Bezüglich des TDM wirkt sich im Allgemeinen eine große Transplantatdimension günstig auf die optischen Qualitäten aus, während eine niedrige Rate immunologischer Abstoßungsreaktionen durch ein kleines Transplantat begünstigt wird. Aus diesem Grund sollte das Transplantat „so groß wie möglich, jedoch so klein wie nötig“ sein [16, 17]. Die Fixierung des Transplantates sollte – wie immer bei ausgedehnten Defekten der Bowman-Lamelle – mit reiner Einzelknüpfnahttechnik erfolgen, um im Falle einer Fadenlockerung einzelne Nähte unproblematisch an der Spaltlampe entfernen zu können.

Bei persistierendem nicht perforiertem Hornhautulkus herpetischer Genese sollte jedoch eine AMT frühzeitig erwogen werden, um eine Keratoplastik à chaud primär zu vermeiden [18]. Bei tiefem Ulkus bis hin zur nicht epithelialisierten Descemetozele bietet sich z. B. eine Triple-Graft-Sandwich-AMT (= 3-mal Graft und 1‑mal Patch) zur Stabilisierung der Augenoberfläche an [18, 19]. Die Keratoplastik à chaud sollte bei Herpes im Zeitalter der AMT auf die Perforation beschränkt bleiben. Ansonsten erlaubt die AMT (typischerweise als „Multigraft-Sandwich“) im Akutstadium, die optische Keratoplastik in das reizfreie und damit prognostisch wesentlich günstigere Intervall zu postponieren [1, 17, 19].

Bekanntlich hat die AMT zahlreiche positive Eigenschaften bei Erkrankungen der Augenoberfläche, darunter mechanischer Schutz, Reepithelisierung und Reduzierung von Entzündungen [18].

Am Schluss ist es wichtig, darauf hinzuweisen, dass die Polymerasekettenreaktion(PCR)-Untersuchung auf HSV-DNA sowie die histologische und elektronenmikroskopische Untersuchung von Hornhautbiopsaten nach Keratoplastik bei der Identifizierung von Erregern, der Diagnosestellung und der Behandlung des Patienten mit HSV-Keratitis eine große Hilfe sein können [20].

Studien zeigen, dass sowohl eine Entzündung als auch potenziell replizierfähige HSV-Partikel länger in der Hornhaut persistieren als klinisch erkennbar und dass dieser Erkenntnis postoperativ Rechnung getragen werden muss [20]. Deswegen ist in Fällen, bei denen der Verdacht auf eine herpetische Genese besteht, die systemische und topische Acyclovir-Therapie die Conditio sine qua non für die Prognose und Prävention rezidivierender, herpetischer Augenkrankheiten [21]. Eine orale, antivirale Therapie kann die Rezidivrate der ulzerierenden nekrotisierenden stromalen Keratitis verringern [1, 21].

## Schlussfolgerungen

Histoacryl-Kleber zur Behandlung der Hornhautperforation herpetischer Genese stellt eine Behandlungsoption in der Akutphase dar. Histoacryl-Kleber bietet eine tektonische Unterstützung und bewahrt die strukturelle Integrität des Bulbus mit dem Verständnis, dass im reizfreien Zustand eine elektive optische PKP zur visuellen Rehabilitation erforderlich sein wird. Die Verzögerung der PKP durch den Einsatz von Hornhautkleber führt in der Regel nicht zu schlechteren Ergebnissen.
